# Practice Variation in Spontaneous Breathing Trial Performance and Reporting

**DOI:** 10.1155/2016/9848942

**Published:** 2016-03-29

**Authors:** Stephanie Godard, Christophe Herry, Paul Westergaard, Nathan Scales, Samuel M. Brown, Karen Burns, Sangeeta Mehta, Frank J. Jacono, Dalibor Kubelik, Donna E. Maziak, John Marshall, Claudio Martin, Andrew J. E. Seely

**Affiliations:** ^1^University of Ottawa Medical School, 451 Smyth Road, Ottawa, ON, Canada K1H 8M5; ^2^Ottawa Hospital Research Institute, 725 Parkdale Avenue, Ottawa, ON, Canada K1Y 4E9; ^3^The Ottawa Hospital, University of Ottawa, 501 Smyth Road, Ottawa, ON, Canada K1H 8L6; ^4^Intermountain Medical Center, University of Utah, 5121 Cottonwood Street, Murray, UT 84157, USA; ^5^St. Michael's Hospital, University of Toronto, 30 Bond Street, Toronto, ON, Canada M5B 1W8; ^6^Mount Sinai Hospital, University of Toronto, 600 University Avenue, Toronto, ON, Canada M5G 1X5; ^7^University Hospitals Case Medical Center and Louis Stokes Cleveland VA Medical Centre, Case Western Reserve University, 11100 Euclid Avenue, Cleveland, OH 44106, USA; ^8^London Health Science Centre, Victoria Hospital, Western University, 800 Commissioners Road E., London, ON, Canada N6A 4G5

## Abstract

*Background*. Spontaneous breathing trials (SBTs) are standard of care in assessing extubation readiness; however, there are no universally accepted guidelines regarding their precise performance and reporting.* Objective*. To investigate variability in SBT practice across centres.* Methods*. Data from 680 patients undergoing 931 SBTs from eight North American centres from the Weaning and Variability Evaluation (WAVE) observational study were examined. SBT performance was analyzed with respect to ventilatory support, oxygen requirements, and sedation level using the Richmond Agitation Scale Score (RASS). The incidence of use of clinical extubation criteria and changes in physiologic parameters during an SBT were assessed.* Results*. The majority (80% and 78%) of SBTs used 5 cmH_2_O of ventilator support, although there was variability. A significant range in oxygenation was observed. RASS scores were variable, with RASS 0 ranging from 29% to 86% and 22% of SBTs performed in sedated patients (RASS < −2). Clinical extubation criteria were heterogeneous among centres. On average, there was no change in physiological variables during SBTs.* Conclusion*. The present study highlights variation in SBT performance and documentation across and within sites. With their impact on the accuracy of outcome prediction, these results support efforts to further clarify and standardize optimal SBT technique.

## 1. Introduction

Physicians in the Intensive Care Unit (ICU) are challenged with difficult decisions regarding when and how to liberate critically ill patients from mechanical ventilation. Weaning, or the reduction of ventilatory support in preparation for extubation, is initiated as early as possible to avoid the morbidity associated with prolonged ventilation [1–3]. Clinicians aim to balance the benefits of early extubation with the risk of failed extubation and reintubation, which is associated with higher mortality and long-term disability [4–6]. Minimizing the duration of mechanical ventilation while optimizing the chance of successful extubation [7] requires reliable strategies to predict failure in at-risk patients [4, 8] and is one of the most important challenges faced by clinicians in caring for critically ill patients.

In response to recognized inconsistencies surrounding the weaning and extubation process, evidence and opinion-based guidelines [9–11] have been published. These documents offer recommendations for management at each step of liberation, including the use of spontaneous breathing trials (SBTs) to test extubation readiness [12]. To expedite weaning, many centres have independently adapted these guidelines into site-specific protocols, leading to heterogeneous implementation of SBTs across centres [12, 13]. Despite widespread adoption [9, 10, 12–14], SBT techniques have not been rigorously defined, with no clear consensus regarding how they should be performed in clinical practice [12, 13].

The literature reveals significant variation in the implementation of existing guidelines into clinical practice. There is consensus on the use of low levels of ventilator support [9, 10, 13, 15, 16] and strong agreement for minimizing sedation [6, 9, 17–21], though precise targets have not been formally articulated [9, 10]. Typical clinical criteria assessed to determine patient readiness encompass patients meeting numerous objective and subjective parameters [22]. The physiologic variables traditionally used to determine SBT tolerance are measured at various time points during the course of an SBT, with no agreement on ideal timing of reporting [4, 6, 13, 14, 23–26]. While a separate issue, the uncertainty in predicting extubation outcomes is highlighted by the lack of clarity regarding which patients may benefit from early tracheotomy [27].

Our principal objective was to investigate and describe inter- and intrainstitutional practice variation in the implementation of SBTs, with regard to levels of ventilator support, oxygenation, and sedation scores during which SBTs are performed, as well as subjective clinical criteria reported in determining outcome. A secondary objective was to evaluate trends in physiologic variables over the course of an SBT to better understand effective timing of measurement and reporting.

## 2. Material and Methods

### 2.1. Study Design and Setting

This study is a prospective observational study analyzing the techniques used for SBTs across 8 ICUs in North America. SBT data was obtained from the Weaning and Variability Evaluation (WAVE) study [28], a multicentre study that investigated variability analysis during SBT to predict extubation outcomes. All SBTs from patients enrolled in the original WAVE study were included in this study. The study was approved by the Research Ethics Boards at each participating site; the need for informed consent was waived as the study was observational, and data were deidentified.

### 2.2. Data Collection

Respiratory Therapists (RTs) performed SBTs at a frequency and duration determined by the treating team in this observational study, though the protocol recommended at least a 30-minute trial. They completed Case Report Forms (CRFs) documenting data on ventilator settings [pressure support (PS), positive end expiratory pressure (PEEP), fraction of inspired oxygen (FiO_2_), and physiologic variables including heart rate (HR), respiratory rate (RR), tidal volume (TV), O_2_ saturation, and the rapid shallow breathing index (RSBI) (TV/RR)] and sedation level [Richmond Agitation Scale Score (RASS) or equivalent]. The protocol recommended a maximum of 7 and 5 cmH_2_O for PS and PEEP, respectively, during SBT, in keeping with guidelines, but no further protocolization was required. The extubation CRF collected the subjective readiness criteria chosen for assessment from a predefined checklist: good spontaneous cough, good cough with suctioning, gag reflex, head lift, firm hand grip, obeys commands, cuff leak, pain controlled, neurologically intact, hemodynamically stable, reversal of indication for ventilation, negative fluid balance for 24 hours, good urine output (over 4 hours), and absence of sedative infusion. RTs were asked to document which subjective clinical criteria were assessed in order to determine patient readiness for extubation. Forms were to be filled out at the time of the SBT prior to extubation.

### 2.3. Data Analysis

SBT data was extracted from CRFs and organized into (1) SBT performance variables (PEEP/PS, FiO_2_, and RASS at which an SBT was conducted) and (2) SBT reporting (subjective extubation criteria and physiologic variables measured and reported over the course of an SBT). Value ranges were predefined for each variable: PS/PEEP (<5, 5, 6–10, and >10 cmH_2_O); FiO_2_ (21–25, 26–30, 31–35, 36–40, and >40%); sedation (RASS: lightly sedated (−2), drowsy (−1), alert and calm (0), restless (1), and agitated (2)).

For analysis of SBT performance, the distributions of ventilator settings, FiO_2_ levels, and RASS were described in two ways: (1) collectively amongst all 931 SBTs, representing the distributions in our study population, and (2) within each of the 8 sites, representing the centre-specific distributions. Mean SBT durations ± standard deviation were calculated for all SBTs. In calculating proportions, the denominator was expressed as the number of SBTs with available data for each analysis, as few did not have complete data from CRFs.

For analysis of SBT reporting, each subjective criterion was evaluated for per-centre incidence of use (number of times a criterion was assessed by RTs/number of patients at site). Physiologic variables (HR, RR, TV, and O_2_ sat) were averaged at each time point during an SBT (2, 15, and 30 minutes) and plotted to depict any trends that may speak to the ideal timing of measurement and reporting. The overall mean ± standard deviation and coefficient of variation were calculated for each variable. The range was calculated per SBT (maximum − minimum value) and averaged to illustrate the average change in physiologic variable over the course of an SBT.

For analysis of SBT outcome, we calculated the success rate across all 931 SBTs based on the outcome (pass, fail, or equivocal) documented in the SBT CRF. We then calculated the portion of passed SBTs (*n* = 734) that were not followed by an attempt at extubation, the frequency of this across sites, and the distribution of RASS in these patients.

## 3. Results

**Figure 1:**
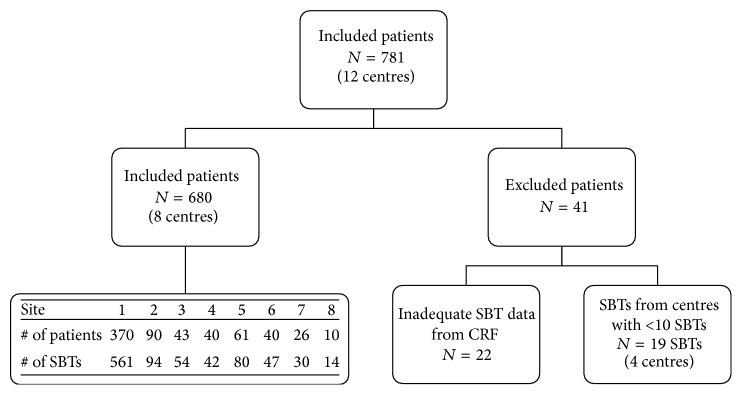
Flow diagram of selection of patients. The diagram shows how the dataset was reduced to ensure only spontaneous breathing trials (SBTs) with adequate case report form (CRF) data were included, and only sites with >10 SBTs were compared for analysis. It includes a breakdown of number of patients and SBTs across sites. All SBTs from all patients enrolled in the Weaning and Variability Evaluation study were originally included.

The WAVE study enrolled 721 patients across 12 North American sites, of which 22 patients were excluded due to inadequate SBT data from CRFs. Data from 4 centres that contributed fewer than 10 SBTs were excluded, leaving 8 ICUs (7 in Canada, 1 in the United States) with data on 680 patients and 931 SBTs (Figure 1). Of these, 502 patients (74%) had a single SBT captured at their time of enrolment and 178 (26%) patients had data on multiple SBTs (70% had 2, 18% had 3, 5% had 4, 3% had 5, 1.7% had 6, 1.1% had 7 SBTs, and 0.6% had over 16 SBTs performed). Patients with a single SBT may have had multiple SBTs performed prior to enrolment or some SBTs may not have been recorded for technical reasons. The breakdown of patients and SBTs across sites can be seen in Figure 1. Sites 1 and 2 were separate ICUs in a single institution, accounting for 68% of patients and 70% of SBTs. The average age of enrolled patients was 62.7 years, with 49% male and a median age of 64 (maximum age 92, minimum age 18).

### 3.1. SBT Performance

SBTs were performed for a mean duration of 38 ± 18 minutes. 8.5% of SBTs were terminated before the 30-minute mark, with 4.9% lasting less than 20 minutes and 1.1% less than 10 minutes. Reasons for early termination were as follows: 51% for respiratory compromise, 26% for cardiovascular compromise, 15% for agitation, 6% for increased secretions, and 2% for decreased oxygen saturation.

Ventilator settings prior to the SBT predominately ranged from 6 to 10 cmH_2_O for both PEEP and PS. Most SBTs (~80%) were conducted with 5 cmH_2_O PEEP and PS (Table 1). Variability in ventilator settings across centres is shown in Table 2. During SBTs, sites 1–3 almost exclusively use 5 cmH_2_O PS and PEEP, while sites 5, 6, and 8 preferentially used 0 cmH_2_O and sites 4 and 7 employed a mixture of PEEP/PS settings. Sites 1–3 almost exclusively used a 5/5 PEEP/PS combination, while others demonstrate alternative settings (Table 2).

**Figure 2:**
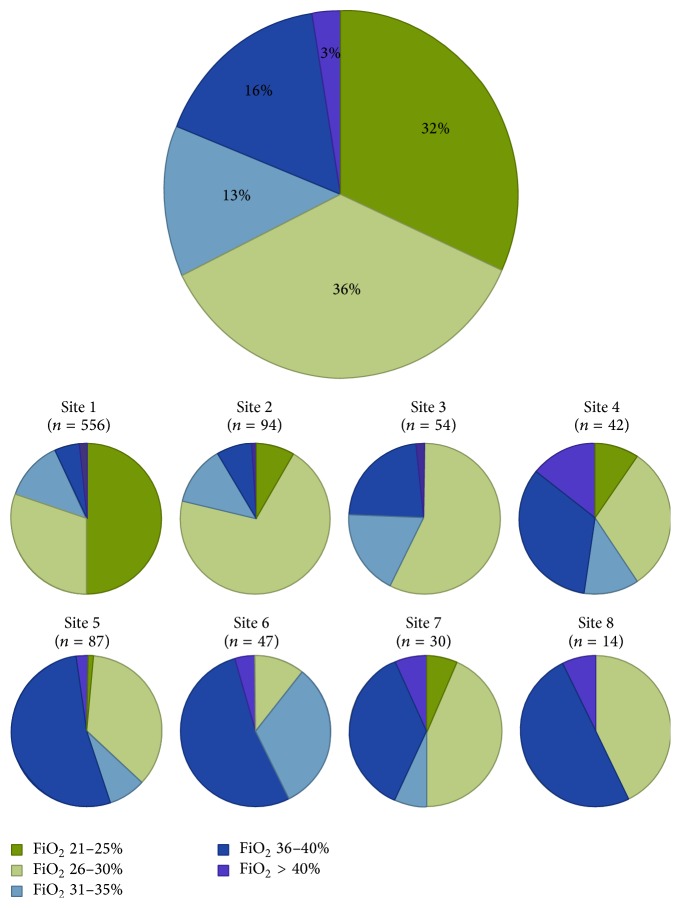
Distribution of fraction of inspired oxygen (FiO_2_) ranges overall and across sites. The large pie graph in this figure depicts the overall distribution in FiO_2_ values from all spontaneous breathing trials (SBTs) with available data for oxygenation. The series of small pie graphs represent the centre-specific distributions, allowing comparison across sites. Values are expressed as % proportion of SBTs. *N* values represent the number of SBTs at each site with available data.

Nearly all FiO_2_ values matched those of pre-SBT ventilator settings and the majority (68%) were performed at FiO_2_ 21–30%, with significant variability across sites (Figure 2). SBTs performed at FiO_2_ > 40% ranged in frequency from 1 to 14% across sites.

**Figure 3:**
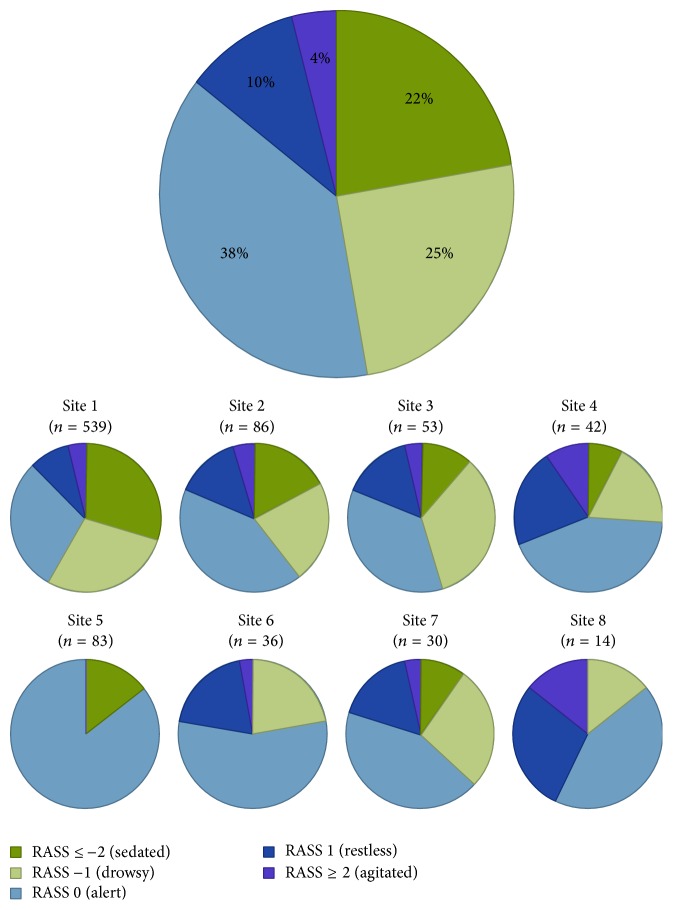
Distribution of Richmond Agitation Scale Score (RASS) ranges overall and across sites. The large pie graph in this figure depicts the overall distribution in RASS scores from all spontaneous breathing trials (SBTs) with available data for sedation. The series of small pie graphs represent the centre-specific distributions, allowing comparison across sites. Values expressed as % proportion of SBTs. *N* values represent the number of SBTs at each site with available data.

While 38% of SBTs were performed in patients with a RASS of 0, Figure 3 illustrates that the majority were performed at nonzero levels of sedation, 22% of which were at RASS ≤ −2 indicating a sedated patient. There was notable variation across sites, with RASS 0 ranging from 29% to 86% and RASS ≤ −2 ranging from 0% to 29%.

### 3.2. SBT Reporting

Table 3 depicts the incidence of use of each subjective clinical extubation criterion across sites. “Obeys commands” and “hemodynamically stable” were most frequently assessed (71 ± 11%, 67 ± 11%, resp.). The top four most frequently sited criteria were separated by a small margin (6%). Criteria were employed heterogeneously across centres, with the assessment of “cuff leak” ranging from 13 to 72% SBTs (mean 51 ± 21%) and “urine output” from 0 to 54% (mean 36 ± 18%).

**Figure 4:**
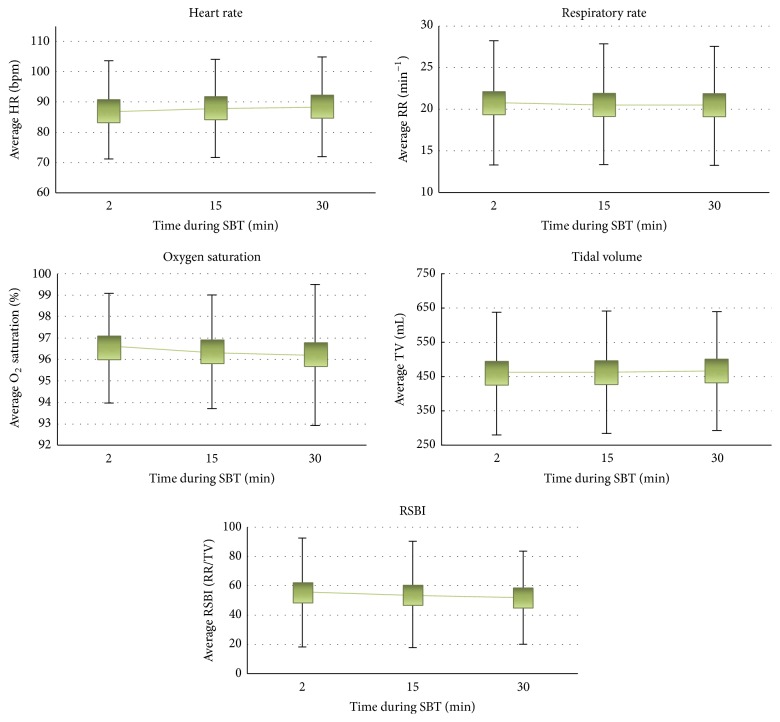
Average physiological variables (heart rate [HR], beats/min; respiratory rate [RR], breaths/min; tidal volume [TV], mL; O_2_ saturation, %) and rapid shallow breathing index (RSBI), RR/TV. This series of figures depict the change in average physiological variable over three time points (2 min, 15 min, and 30 min). Averages were calculated across all spontaneous breathing trials (SBTs) with available data at each time point. Error bars represent 1 standard deviation.

Physiologic variables displayed relatively wide ranges (Table 4); however neither the physiological variables (HR, RR, TV, and O_2_ sat) nor RSBI measured changed appreciably over the 3 time points during an SBT (2, 15, and 30 minutes), as seen in Figure 4.

### 3.3. SBT Outcome and Extubation Rates

The percentage of successful SBTs (i.e., a patient passed the SBT according to site-specific criteria) was 79% (734 out of 931 SBTs), 11% were equivocal, 8% were not successful, and 2% had missing information. 20% (150 of 734) of SBTs deemed successful did not result in an immediate attempt at extubation. For sites 1, 3, 5, and 8, the proportion of patients who successfully passed an SBT but were not extubated ranged from 11% (site 5) to 24% (site 1). All other sites had rates lower than 5%. There did not seem to be any relation to the level of sedation, as the RASS of these 150 SBTs were more or less equally distributed between −3 and 1.

## 4. Discussion

This study evaluated and described SBT performance and reporting in 931 SBTs from 680 patients across 8 North American ICUs. The majority of SBTs (80% and 78%) were performed at PEEP and PS of 5 cmH_2_O, with 5/5 cmH_2_O being the most common setting. Most SBTs (68%) were conducted at FiO_2_ 21–30%. The majority of SBTs (47%) were performed at a RASS < 0, with 22% at RASS ≤ −2. The choice of subjective clinical criteria was heterogeneous with differences in use of up to 60% across sites. The trend of physiologic variables did not change over the course of SBT. SBTs were performed at a mean duration of 38 + 18 minutes.

The overall predominance of 5/5 cmH_2_O PEEP/PS reflected the practice at four sites, including the centre that contributed 68% of patients to the study, likely underestimating centre variation in ventilator settings. Nevertheless, variation was apparent in that some sites adhered to PEEP and/or PS of 0 cmH_2_O in various combinations, while others displayed no clear preference for SBT settings. This variability may have important implications for extubation practice and reflects the controversy over “minimal” versus no ventilatory support during SBTs. While previous studies have argued against T-piece SBTs [29] or found no difference in outcome [9–11], others have advocated for zero support, suggesting that low levels of support overestimate the patient's ability to handle the respiratory load after extubation [15]. Patient characteristics, such as obesity, COPD, or the size of endotracheal tube, may also influence choice of ventilatory support [10, 15], warranting further research [13]. Last, differences in ventilator settings impact breathing pattern variability (BPV), a novel method evaluated to predict extubation outcome [16, 28]. While the literature unanimously supports low level ventilation [9, 10, 13], there is ongoing debate over its precise definition and the exact levels to be used in differing patient conditions.

The majority of SBTs were performed at a nonzero RASS, RASS 0 reflecting the calm and alert patient state supported by the literature, and a surprising one-fifth proportion (22%) of SBTs were performed in patients with RASS ≤ −2, with several implications regarding extubation. Oversedation increases risk of prolonged ventilation and adverse outcomes [18, 19] and suppresses heart rate and respiratory rate variability [28] and clinical tools used to predict extubation outcome, thereby hampering accuracy. On the other end of the spectrum, interpretation of clinical criteria used in extubation outcome prediction may be skewed in agitated patients (positive RASS). Our findings are inconsistent with evidence supporting minimal sedation, as well as recent initiatives advocating incorporating sedation minimization strategies into weaning protocols [22, 30]. Studies recommend the reduction of psychoactive medication [17] and trials of minimal sedation while performing SBTs [6, 20, 21, 30].

The majority of FiO_2_ levels were below the recommended 40% and the variation noted is not clinically relevant as it reflects diversity in pre-SBT oxygen requirement, suggesting little role for the standardization of this parameter in protocols. Nevertheless, the variation in oxygenation reinforces the overall heterogeneity of the patients in the study and possibly SBT oxygenation practice noted in this study.

Our findings depict the diversity of subjective clinical criteria assessed to inform the decision-making process surrounding extubation readiness. Criteria were reported heterogeneously across and within sites and no criteria prevailed as most or least common, demonstrating a lack of standardized approach to patient assessment. Inconsistency in assessing clinical criteria diminishes the reproducibility of outcome prediction, which may be further exacerbated by human error inherent in interpreting subjective parameters [13, 22]. Furthermore, studies comparing the predictive performances of these parameters, such as cough strength and fluid balance [31, 32], are limited by practice variation across sample populations [22]. While variability in choice of criteria may reflect patient's clinical status [33, 34], further investigation is needed to determine the value of patient-specific criteria. Overall, our findings highlight the ambiguity surrounding how to use clinical criteria to assess extubation readiness.

Measurement and reporting of vital signs have traditionally been utilized to determine extubation readiness by evaluating patient tolerance of SBTs. While the literature offers opinions on when and how often physiologic variables and RSBI should be documented, our findings reveal that, on average, their values do not change appreciably over the course of an SBT.

While the purpose of this study is not to correlate SBT technique with extubation outcome, we included a brief analysis of SBT outcome. The majority of successful SBTs resulted in an attempt at extubation; however 20% were not and instead followed by additional trials, again with variation across sites. We did not gather rationale on why these SBTs did not result in extubation, though one can presume it is based on clinical gestalt surrounding patient trajectory, weighing the risks and benefits of prolonging ventilation.

There are several limitations to this study. First, we could not provide meaningful analysis of SBT technique and extubation failure rates given the statistically uncommon extubation failure rate (although 13% is likely too high clinically). The number of patients in each subgroup was too small; for instance, the number of final SBTs performed with T-piece (PEEP/PS = 0) was 37 (5.5% of SBTs). However, our main objective was to describe practise variation and, to our knowledge, this is the largest multicentre study of its kind addressing the fundamental issue of inconsistency in SBT practice. As a second limitation, the WAVE study was predominately obtained from a single centre contributing 68% of patients (70% of SBTs); however, practice variation would likely increase with more representation from other centres as ventilator support during SBTs in particular remains highly controversial. Additionally, 7 of the 8 centres were in Canada, where RTs are primarily responsible for the weaning process, which may affect variation not observed elsewhere. Third, we did not collect data on the use of sedation medication and its implications on RASS. We also did not gather information on how RTs selected extubation criteria and it is possible that some were assessed informally without documentation. How intensive care clinicians interpreted these criteria in their decision-making process and whether variability represents centre or physician-specific preferences are unknown. Fourth, while many of the centres in this study may have some internal standards for SBT technique, we did not formally evaluate the existence of protocols across sites and it was out of the scope of this study to analyze any discrepancy between SBT performance and reporting. Furthermore, the existence of pre-SBT readiness screening was not assessed, and this may impact SBT outcome and variability. Nevertheless, the practice variation noted in this study reflects overall variability in content of and adherence to any site-specific protocols. Fifth, our study included single SBTs that may have been preceded by multiple trials prior to enrolment. While this may introduce bias in SBT and extubation outcomes, the objective of this study is to describe SBT technique overall, irrespective of weaning stage. Sixth, we assumed that FiO_2_ was titrated appropriately for all patients and we did not record PaO_2_ directly as a measure of oxygenation. Last, a limitation in trending only average physiologic variables is that individual patients were not graphed over time, some of which may have shown variation over SBT. However, isolated trends in vital signs would not be enough to suggest ideal time points of measurement for the purpose of standardization.

The benefits of generalized weaning protocols have been demonstrated in controlled trials [13, 17, 19, 35–37] and attention should be shifted toward standardizing SBT technique [17]. Efforts to improve persistent extubation failure rates have focused on developing objective predictive indices, though they have proven unreliable to date [8, 10, 17, 23, 32]. Standardizing SBT technique will help power future studies on extubation outcome by alleviating coefficients of variation [17, 22] and will improve adherence to evidence-base practice [13]. However, SBT protocols are intended to act as a process guide rather than a one-size-fits-all approach, with patient-specific adaptation by physicians.

Given the practice variation observed in this study, further research is needed to correlate SBT technique with outcome and determine optimal targets for standardization of SBT performance and reporting. For example, zero-support ventilation (PEEP/PS) and minimization of sedation may improve accuracy of outcome prediction and variability analysis. Little is known regarding the utility of various extubation readiness criteria. As we did not detect any meaningful change on average in vital signs or TV during an SBT, the precise timing of recording physiological parameters does not appear to be critical. While not evaluated in this study, the interaction between SBT protocols, results and interpretation, and a clinician's decision-making process is fertile ground for further investigation in order to augment safety of extubation in critically ill patients.

## Figures and Tables

**Table 1:** Overall distribution of PEEP and PS ranges amongst all SBTs. Ranges shown for pre- and during-SBT levels.

Range (cmH_2_0)	PEEP		PS	
	Pre-SBT	During-SBT	Pre-SBT	During-SBT
0 to 4	2%	14%	5%	8%
5	22%	80%	5%	78%
6 to 10	75%	6%	74%	13%
>10	1%	0%	16%	1%

Values expressed as % proportion of all SBTs. PEEP, positive end expiratory pressure; PS, pressure support.

**Table 2:** Distribution of PS and PEEP ranges and preferred setting combinations (PEEP/PS).

Range (cmH_2_O)	Site 1 (*n* = 561)	Site 2 (*n* = 94)	Site 3 (*n* = 54)	Site 4 (*n* = 42)	Site 5 (*n* = 89)	Site 6 (*n* = 47)	Site 7 (*n* = 30)	Site 8 (*n* = 14)
PS								
0 to 4	0%	0%	0%	2%	7%	98%	39%	100%
5	93%	97%	100%	90%	1%	2%	52%	0%
6 to 10	5%	1%	0%	7%	92%	0%	9%	0%
>10	1%	2%	0%	0%	0%	0%	0%	0%
PEEP								
0 to 4	0%	1%	0%	0%	94%	85%	9%	7%
5	94%	98%	98%	76%	2%	15%	70%	86%
6 to 10	6%	1%	2%	24%	3%	0%	22%	7%
>10	0%	0%	0%	0%	0%	0%	0%	0%
Preferred settings								
PEEP/PS	5/5 (92%)	5/5 (96%)	5/5 (98%)	5/5 (69%)	0/6 (90%)	0/0 (85%)	5/5 (27%)	5/0 (86)%
(% incidence)				8/5 (21%)			5/0 (23%)	

Values expressed as % proportion of spontaneous breathing trials (SBTs) unless otherwise specified, that is, % incidence of preferred settings. PEEP: positive end expiratory pressure; PS: pressure support. *N* values represent the number of SBTs at each site with available data.

**Table 3:** Distribution of clinical extubation criteria used across sites and average number of criteria assessed per site.

Extubation criteria	% incidence of use									
	Average	Stdev	Site 1 (*n* = 309)	Site 2 (*n* = 90)	Site 3 (*n* = 43)	Site 4 (*n* = 40)	Site 5 (*n* = 61)	Site 6 (*n* = 40)	Site 7 (*n* = 26)	Site 8 (*n* = 10)
Obeys commands	**71%**	**11%**	76%	57%	70%	80%	70%	53%	81%	80%
Hemodynamically stable	**67%**	**11%**	74%	57%	70%	80%	74%	45%	69%	70%
Gag reflex	**67%**	**8%**	74%	68%	53%	78%	72%	63%	65%	60%
Neurologically intact	**65%**	**10%**	69%	61%	67%	70%	69%	45%	77%	60%
Good spontaneous cough	**60%**	**11%**	69%	63%	53%	63%	64%	55%	77%	40%
Good cough suctioning	**60%**	**9%**	76%	69%	47%	63%	61%	60%	58%	50%
Pain controlled	**55%**	**11%**	71%	58%	44%	63%	44%	53%	65%	40%
No more indication	**53%**	**14%**	66%	57%	53%	60%	46%	23%	62%	60%
Hand grip	**52%**	**12%**	64%	41%	60%	55%	38%	50%	69%	40%
Cuff leak	**51%**	**21%**	72%	54%	51%	45%	72%	13%	35%	70%
No sedative infusion	**50%**	**9%**	53%	34%	44%	50%	62%	45%	62%	50%
Head lift	**43%**	**15%**	61%	41%	56%	45%	49%	40%	38%	10%
Urine output	**36%**	**18%**	54%	50%	37%	48%	33%	23%	46%	0%
Negative fluid balance	**31%**	**9%**	45%	32%	30%	23%	28%	28%	42%	20%
Average # of criteria used/Pt			**9**	**7**	**7**	**8**	**8**	**6**	**8**	**7**

Values expressed as % incidence of use per site (number of times a criterion is used per site/total number of criteria used per site). Pt, patient; Stdev, standard deviation; #, number. *N* values represent the number of patients at each site with available data.

**Table 4:** Measures of variance for physiologic variables (tidal volume, respiratory rate, heart rate, and O_2_ saturation). Expressed as mean, standard deviation, coefficient of variation, and average range per spontaneous breathing trial (SBT).

Physiologic variable	Mean value	Stdev	Coefficient of variation	Average range/SBT
Tidal volume (mL)	461.9	176.1	0.38	87.4
Respiratory rate (breaths per min)	20.6	7.3	0.35	3.9
Heart rate (beats per min)	87.9	16.3	0.19	5.8
O_2_ saturation (%)	96.3	2.8	0.03	1.5
